# Use of Cool Drinking Water as a Strategy Under High-Ambient-Temperature Conditions in New Zealand Rabbits: Growth Performance, Carcass Traits and Physiological Responses

**DOI:** 10.3390/vetsci13030262

**Published:** 2026-03-11

**Authors:** Gamaliel Molina-Gámez, Juan C. Robles-Estrada, Jaime N. Sánchez-Pérez, Francisco G. Ríos-Rincón, Jesús J. Portillo-Loera, Juan E. Sánchez-Torres, Horacio Dávila-Ramos

**Affiliations:** 1 Facultad de Medicina Veterinaria y Zootecnia, Universidad Autónoma de Sinaloa, Culiacán 80246, Sinaloa, Mexico; gamalielmolinagamez@gmail.com (G.M.-G.); jaime.sanchez.fmvz@uas.edu.mx (J.N.S.-P.); fgrios@uas.edu.mx (F.G.R.-R.); portillo6422@uas.edu.mx (J.J.P.-L.); 2 Facultad de Medicina Veterinaria y Zootecnia, Universidad Autónoma del Estado de México, Toluca 50090, Estado de México, Mexico; edreie@yahoo.com.mx

**Keywords:** rabbits, heat stress, cool water

## Abstract

Heat stress represents a major challenge in rabbit production in tropical regions, where high temperature–humidity index (THI) values compromise thermal homeostasis and animal welfare. This study evaluated the effect of providing cool drinking water as a heat stress mitigation strategy on growth performance, carcass traits, water intake, and physiological responses in growing New Zealand White rabbits. Sixteen male rabbits were assigned to receive either drinking water at ambient temperature (33.9 ± 1.5 °C) or cooled water (16.7 ± 1.8 °C) supplied during periods of highest thermal load (10:00–17:00 h) over a four-week experimental period. Ambient temperature and relative humidity were monitored to calculate THI, and body temperatures were recorded during morning and afternoon periods. Average daily gain, carcass traits, and water intake were not affected by drinking water temperature (*p* > 0.05). However, the feed-to-gain ratio over the overall experimental period was higher in rabbits receiving cooled water (*p* = 0.03). In contrast, rectal temperature during the afternoon was significantly reduced in rabbits receiving cooled water, as reflected by a significant water × period interaction (*p* = 0.03), representing a 0.62% reduction compared with rabbits receiving normal drinking water, particularly during periods of greater thermal challenge, whereas ear and body surface temperatures were mainly influenced by the experimental period (*p* < 0.01). These results indicate that moderate cooling of drinking water elicits measurable physiological responses associated with short-term thermoregulatory adjustment, without improving growth performance. Providing cool drinking water represents a practical strategy to support thermoregulation under heat stress conditions in rabbit production systems in tropical climates.

## Simple Summary

Rabbits raised in tropical climates are frequently exposed to high temperatures and humidity, which can compromise their ability to regulate body temperature and challenge thermal balance. Providing cool drinking water has been proposed as a simple management strategy to help animals cope with heat stress. In this study, growing rabbits were offered either drinking water at ambient temperature or moderately cooled water during the hottest hours of the day. While daily weight gain, feed intake, carcass traits, and water intake were not affected by water temperature, rabbits receiving cooled water showed lower body core temperature during the afternoon, when heat stress was greatest. However, the feed-to-gain ratio over the overall experimental period was higher in rabbits receiving cooled water. These responses indicate a localized thermoregulatory adjustment without clear productive advantages. These findings suggest that moderate cooling of drinking water can support thermoregulation under heat stress conditions in rabbit production systems in tropical environments.

## 1. Introduction

Heat stress represents one of the main limiting factors in livestock production worldwide, particularly in tropical and subtropical regions, where high temperatures and elevated ambient humidity negatively affect animal productivity. Heat stress is associated with reductions in voluntary feed intake, growth, and efficiency, thereby compromising overall growth performance and meat quality in animal production systems [1].

Rabbit meat represents a relevant alternative as a source of high quality animal protein, characterized by its low fat and cholesterol content and high biological value. In addition, rabbit production is distinguished by rapid productive turnover and high reproductive efficiency [2]. However, rabbits are particularly susceptible to high-ambient temperatures, as they lack functional sweat glands and have a limited capacity to dissipate excess body heat. In this species, evaporation associated with panting and peripheral vasodilation, especially at the auricular level, constitutes the main thermoregulatory mechanism [3,4].

Heat stress induces physiological alterations that hinder the maintenance of thermal homeostasis, which in rabbits are manifested through changes in heart and respiratory rates, electrolyte imbalances, reduced feed intake of up to 28%, and modifications in energy metabolism [3,5]. These alterations may translate into decreases in weight gain and feed efficiency of up to 25% and 15%, respectively, as well as increased mortality under severe heat stress conditions of up to 9–12% [6,7]. The optimal ambient temperature range for rabbits is between 15 and 25 °C, with a relative humidity of 55–65%. Heat stress occurs when ambient temperature exceeds 30 °C and becomes critical above 35 °C, at which point rabbits exhibit a limited ability to regulate body temperature [6]. Thermal stress conditions in rabbits are classified using the THI as no, moderate, severe, and very severe stress categories [8,9].

Strategies aimed at mitigating the negative effects of heat stress in animals can be grouped into those focused on reducing metabolic heat production, improving body heat dissipation, or increasing heat tolerance through genetic selection [10]. In rabbits, the main measures include improvements in housing infrastructure, such as ventilation and shading, as well as complementary strategies, including nutritional supplementation and the use of low-temperature drinking water as a management approach to facilitate thermoregulation [6].

The use of cool water represents a management strategy for mitigating heat stress in rabbits, either through the provision of drinking water or via direct physical contact [11]. On the one hand, conductive cooling achieved by placing cold water bottles inside the cage significantly reduces respiratory rate and rectal temperature while increasing feed intake [12]. Complementarily, supplying cooled drinking water during periods of peak heat load facilitates internal thermoregulation, which translates into improved weight gain and feed efficiency [13].

Although various strategies to mitigate heat stress in rabbits have been evaluated, most studies have been conducted under climatic or management conditions different from those prevailing in dry tropical regions. These environments, characterized by high temperature–humidity index values and marked daily thermal variation, may influence rabbits’ physiological and productive responses. In Mexico, rabbit production has gained interest as an alternative livestock system in warm regions; however, locally generated experimental evidence supporting management decisions under these environmental conditions remains limited. Therefore, the objective of the present study was to evaluate the effect of providing moderately cooled drinking water during periods of greatest thermal load on growth performance, carcass traits, and physiological responses of growing New Zealand White rabbits raised under dry tropical conditions.

## 2. Materials and Methods

### 2.1. Study Area and Climate Data

This study was conducted at the Rabbit Experimental Unit of the Faculty of Veterinary Medicine and Zootechnics, Autonomous University of Sinaloa, Culiacán, Sinaloa, México (24.77200° N, 107.35439° W). The experiment was conducted in a rectangular open-sided rabbit housing facility measuring 4.5 m in width and 17 m in length. The building was fully roofed, with a central height of 4.0 m and 2.1 m at both lateral ends. The side walls were equipped with curtain systems that allowed natural ventilation and passive natural lighting through the lower section of the structure. Ambient temperature and relative humidity were recorded in situ six times per day at 10:00, 11:30, 13:00, 14:30, 16:00, and 17:00 h throughout the experimental period. Records obtained at 10:00 and 11:30 h were considered morning measurements, whereas those obtained at 13:00, 14:30, 16:00, and 17:00 h were considered afternoon measurements. The THI was calculated using the equation described by [11]: THI = db°C − {(0.31 − 0.31 × RH) × (db°C − 14.4)}, where db°C is the dry-bulb temperature expressed in degrees Celsius and RH is the relative humidity expressed as a decimal fraction. THI values were classified as follows: no heat stress (<27.8), moderate heat stress (27.8–28.9), severe heat stress (28.9–30.0), and very severe heat stress (>30.0) [11].

### 2.2. Animal Management and Treatments

The rabbits used in the experiment were obtained from the breeding population maintained at the same research center. All animals were growing males of similar age, six weeks old, clinically healthy, and raised under identical conditions. From the available population, the sixteen rabbits included in the study met the health criteria and initial body weight range required for inclusion in the experiment.

Sixteen male New Zealand White rabbits (*Oryctolagus cuniculus*), with an average initial live weight of 1429 ± 208 g, were used in this study. Rabbits were allocated to treatments using a randomized complete block design, in which initial live weight was used as the blocking factor to minimize variability among animals. Within each block, rabbits were randomly assigned to one of the two experimental treatments. Rabbits were housed individually in galvanized wire cages measuring 78 cm in length × 48 cm in width × 28 cm in height, equipped with galvanized metal feeders and nipple drinkers. All animals had ad libitum access to a commercial pelleted diet containing 18% crude protein, 14% fiber, and 2.86 Mcal/kg of dry matter of digestible energy. Fresh feed was offered once daily at 08:00 h, allowing a daily refusal margin to ensure voluntary intake and to minimize dust accumulation associated with pelleted diets. Experimental treatments consisted of: (1) drinking water at ambient temperature (33.9 ± 1.5 °C; Normal, control group) and (2) cool drinking water (16.7 ± 1.8 °C; Cool). Cool drinking water was supplied during the hours of highest thermal load (10:00 to 17:00 h).

Drinking water was provided to each rabbit using plastic bottles (capacity: 375 mL) positioned outside each cage and connected to a stainless-steel nipple through an 8 cm tube [14]. In the cooling treatment, bottles were externally covered with expanded polystyrene foam to reduce heat exchange with the environment. At 10:00 h, residual water from the previous day was completely removed to allow calculation of daily water intake. Immediately thereafter, 250 mL of pre-cooled water (13 °C) was added to each bottle and the container was sealed. To maintain reduced water temperature during the period of highest thermal load (10:00–17:00 h), 35 mL of frozen water (ice) was added at 1.5 h intervals (11:30, 13:00, 14:30, 16:00, and 17:00 h), coinciding with environmental measurements. The volume of ice was previously estimated to maintain water temperature within the target range during the daytime heat period. Water temperature was measured directly inside each bottle for both the control and cooling treatments at every scheduled time point (10:00, 11:30, 13:00, 14:30, 16:00, and 17:00 h) using a calibrated digital thermometer.

### 2.3. Growth Performance

Rabbits were weighed on days 1, 8, 15, 22, and 29 of the experiment prior to feed delivery. Feed and water intake were determined by calculating the difference between the amount offered and the refusals. Average daily gain was calculated as the difference between final and initial body weight divided by the number of days in the evaluation period. Feed-to- gain ratio was calculated by dividing daily feed intake by average daily gain [13].

### 2.4. Carcass Traits

Animals were slaughtered following the guidelines of the Mexican Official Standard NOM-033-SAG/ZOO-2014, which defines humane procedures to minimize pain, stress, and suffering. Hot carcass weight was recorded 15–30 min after slaughter, after complete bleeding and removal of the skin, head, distal parts of the fore and hind limbs, and gastrointestinal and urogenital tracts. Carcasses were then chilled for 24 h in a ventilated cold room at 2–4 °C, after which chilled carcass weight was determined. Dressing out percentage was calculated as the ratio between chilled carcass weight and final live weight [15].

### 2.5. Physiological Response

Ear and body surface temperatures were recorded daily at 08:00 and 14:30 h (morning and afternoon periods) using a digital infrared thermometer. Ear temperature was measured at an approximate distance of 5 cm from the central area of the auricular pavilion. Body surface temperature was recorded by aiming the thermometer at the dorsal region between the neck and the loin. Rectal temperature was recorded twice per week using a digital thermometer inserted approximately 2 cm into the rectum. All temperature measurements were performed in triplicate, and the average value was used for statistical analysis [16].

### 2.6. Statistical Analysis

Growth performance and carcass traits variables were analyzed using a randomized complete block design, with initial live weight used as the blocking criterion. Four blocks were formed, each comprising four individually housed rabbits (two rabbits per treatment within each block). For these variables, the experimental unit was defined as the mean of the two rabbits assigned to each treatment within a block. Variables were analyzed using the MIXED procedure of SAS, with treatment included as a fixed effect and block as a random effect. Live weight was analyzed as a repeated measure over time. Dry matter intake, average daily gain, and feed-to-gain ratio were calculated by experimental period (days 1–14, 15–28, and 1–28) and analyzed using mixed models.

Physiological variables, including ear, body surface, and rectal temperatures, as well as water intake, were analyzed using linear mixed models with repeated measures, with the individual rabbit considered the experimental unit. Treatment, period, and their interaction were included as fixed effects, and the individual rabbit was included as a random effect. When significant treatment × period interactions were detected, simple effects were performed to evaluate treatment differences within each period. Statistical significance was declared at *p* ≤ 0.05, whereas values of *p* ≤ 0.10 were considered indicative of statistical trends. Pearson’s correlation coefficients were calculated to explore linear associations between the THI and physiological variables. Correlation analyses were used for descriptive purposes, and statistical significance was declared at *p* ≤ 0.05.

## 3. Results

### 3.1. Environmental Conditions

**Figure 1:**
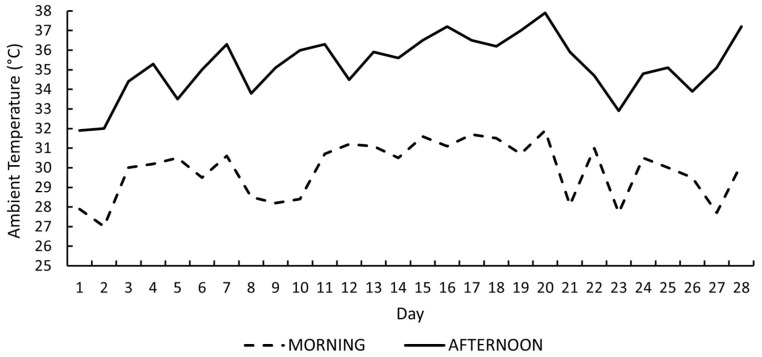
Daily morning and afternoon ambient temperature recorded during the 28-day experimental period.

Daily ambient temperature recorded during the 28-day experimental period is presented in Figure 1. Morning ambient temperature ranged from 27.0 to 31.9 °C, with most values between 29 and 31.5 °C. In contrast, afternoon temperature ranged from 31.9 to 37.9 °C and exceeded 34 °C on 24 of the 28 experimental days. Maximum afternoon values ≥ 37 °C were recorded on seven days, indicating sustained exposure to elevated thermal load during peak heat hours.

**Figure 2:**
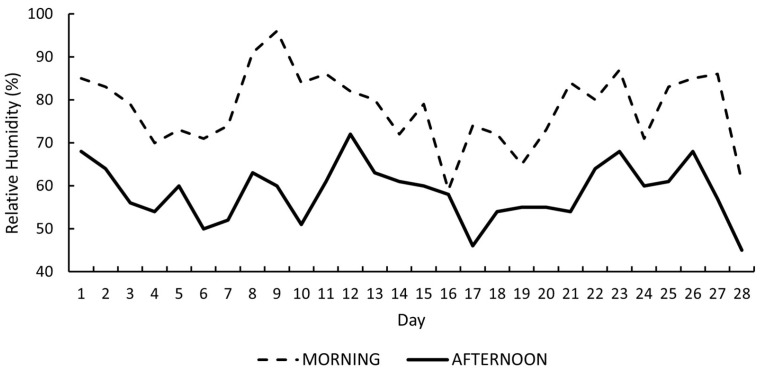
Daily morning and afternoon relative humidity (%) during the 28-day experimental period.

Daily relative humidity recorded during the 28-day experimental period is presented in Figure 2. Morning relative humidity ranged from 59 to 96%, generally exceeding 70% on most days. In contrast, afternoon relative humidity ranged from 45 to 72%, reflecting the typical diurnal decrease associated with rising ambient temperature.

**Figure 3:**
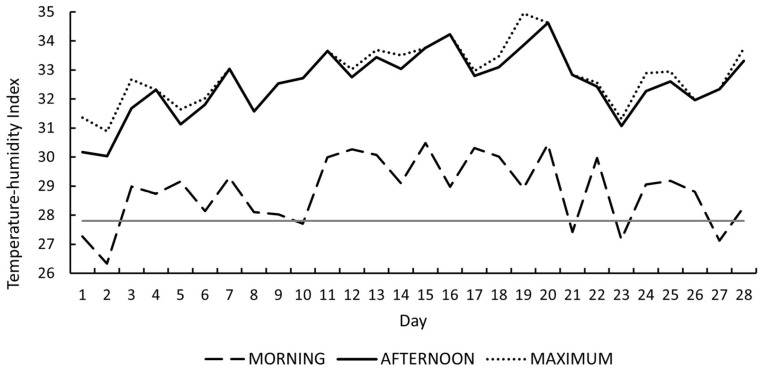
Daily morning, afternoon, and maximum temperature–humidity index (THI) values recorded during the 28-day experimental period. The solid horizontal line represents the heat stress threshold (THI = 27.8) according to [11].

As shown in Figure 3, morning THI ranged from 26 to 30. According to the classification proposed by [11], five days were classified as no heat stress (<27.8), five as moderate heat stress (27.8–28.9), and eighteen as severe heat stress (28.9–30.0). Morning THI did not exceed 30.0 on any day.

In contrast, afternoon THI values ranged from 30 to 35. Twenty-six of the 28 experimental days recorded THI values > 30.0, corresponding to the very severe heat stress category, while two days (THI = 30.0) were classified as severe. Maximum daily THI values ranged from 31 to 35 and were consistently classified as very severe heat stress throughout the entire experimental period. These results confirm sustained exposure of rabbits to severe to very severe heat stress conditions according to the criteria established by [11].

### 3.2. Morning, Afternoon and Maximum THI

The THI values recorded by experimental period are presented in Table 1. Morning THI did not differ significantly among experimental periods (*p* > 0.05) and showed no linear trend over time. In contrast, afternoon THI differed significantly among periods (*p* < 0.01), with the highest values observed during period 3, followed by periods 2 and 4, whereas period 1 showed the lowest values. A significant linear trend across experimental periods was detected for afternoon THI (*p* = 0.02). Maximum THI also differed significantly among periods (*p* < 0.01), with the highest values recorded in period 3. A marginal linear trend was observed for maximum THI over the course of the experiment (*p* = 0.06).

Descriptive analysis (Table 2) showed that normal water exhibited low variability across the experimental period (CV < 5%), indicating stable thermal conditions. In contrast, cooled water presented a higher coefficient of variation.

### 3.3. Growth Performance and Carcass Traits

The effects of drinking water temperature on growth performance traits are presented in Table 3. Drinking water temperature did not affect live weight, dry matter intake, or average daily gain during any of the evaluated periods (*p* > 0.05). Feed-to-gain ratio did not differ between treatments during days 1–14 or 15–28 (*p* > 0.05). However, during the overall experimental period (days 1–28), rabbits receiving normal-temperature drinking water exhibited a lower feed-to-gain ratio compared with those receiving cool drinking water (*p* = 0.03).

The effects of drinking water temperature on carcass traits are shown in Table 4. No significant differences were observed between treatments for hot carcass weight, cold carcass weight, or dressing carcass (*p* > 0.05).

The effects of drinking water temperature on water intake are presented in Table 5. Water intake did not differ significantly between rabbits receiving cool or normal drinking water during any of the experimental weeks (*p* > 0.05).

### 3.4. Physiological and Thermoregulatory Responses

Morning body temperatures are presented in Table 6. In the morning, ear and body temperatures were significantly affected by period (*p* < 0.01), reflecting changes in environmental conditions across weeks, whereas drinking water temperature had no effect on any variable. Rectal temperature remained stable across treatments and periods, indicating that core thermal homeostasis was maintained under morning conditions. A tendency for a Water × Period interaction was observed for body surface temperature (*p* = 0.10), suggesting a period-dependent response. Morning rectal temperature was not affected by water treatment, period, or their interaction.

Afternoon body temperatures are shown in Table 7. During the afternoon, ear and body temperatures were also primarily influenced by period effects (*p* < 0.01), consistent with a strong response to environmental heat load. In contrast, rectal temperature was the only variable affected by drinking water temperature, showing a significant Water × Period interaction (*p* = 0.03) and an overall reduction of approximately 0.62% relative to rabbits receiving ambient-temperature water. Specifically, rectal temperature during Week 4 was approximately 1.14% lower in rabbits receiving cooled drinking water, indicating a treatment-specific effect on core body temperature under higher thermal challenge.

### 3.5. Temperature–Humidity Index and Body Temperature Responses

Pearson’s correlation coefficients between THI and body temperatures recorded during the morning and afternoon are presented in Table 8 and Table 9, respectively. Overall, regardless of treatment, the correlations between THI and the temperature variables showed a consistent pattern across both tables. Ear and body temperatures exhibited the highest correlation magnitudes, whereas rectal temperature showed lower values, indicating a weaker association with the evaluated environmental conditions.

When comparing morning and afternoon measurements, similar patterns were observed; however, during the afternoon, rectal temperature tended to exhibit lower correlations with THI, and even negative values in some periods compared with the morning, indicating a reduced dependence of central temperature on environmental conditions at this time of day relative to peripheral temperatures.

When considering the effect of treatments on rectal temperature, the provision of cool drinking water was associated with lower correlation values with THI, particularly during the morning in periods 2 and 4, as well as during the afternoon in period 2. In contrast, ear and body temperatures maintained associations of similar magnitude with THI, regardless of treatment.

## 4. Discussion

The higher coefficient of variation observed for cooled drinking water reflects the inherent dynamics of the cooling system and should be interpreted as a characteristic of thermal management rather than measurement error. In contrast, normal drinking water showed low variability, indicating stable conditions driven by the ambient temperature.

### 4.1. Growth Performance, Carcass Traits and Water Intake

In the present study, providing cool drinking water during the hours of highest thermal load did not result in improvements in live weight, dry matter intake, or average daily gain. These findings indicate that, under the environmental conditions evaluated, this mitigation strategy did not enhance growth performance despite the exposure of rabbits to heat stress conditions. As demonstrated by the environmental characterization (Figure 1 and Figure 3), afternoon ambient temperature consistently exceeded 34 °C and reached up to 37.9 °C, and afternoon THI values were classified as very severe heat stress (≥30.0) on all 28 days of the experimental period. Therefore, the absence of improvements in growth performance cannot be attributed to an insufficient thermal challenge.

Previous studies have reported improvements in growth performance when cool drinking water was supplied to rabbits exposed to heat stress; however, these responses have been highly variable and strongly dependent on experimental conditions. Positive effects on average daily gain and feed efficiency have been reported when water temperatures between 10 and 15 °C were provided under severe heat stress conditions [11,17]. In contrast, other studies have shown neutral or even negative effects of cooled drinking water on productive performance, particularly when lower water temperatures were used or when animals were evaluated during sensitive physiological stages such as early growth or reproduction [14,18,19].

In the present study, although average daily gain and dry matter intake were not affected by treatment, rabbits receiving cool drinking water exhibited a higher feed-to-gain ratio over the overall experimental period (days 1 to 28) compared with those receiving water at normal-temperature. This response suggests that, under the heat stress conditions of this experiment, the physiological adjustments elicited by cooled drinking water may not translate into improved feed efficiency. Previous authors have reported that the feed-to- gain ratio of rabbits consuming cold water was higher than that of rabbits consuming water at higher temperatures [19]. Under these circumstances, the contribution of cooled drinking water appears to be limited to supporting thermoregulatory balance rather than directly enhancing growth efficiency. Carcass traits in rabbits largely reflect cumulative growth responses and nutrient partitioning over time; therefore, in the absence of sustained differences in feed intake or weight gain, major alterations in carcass composition are not expected.

In the present study, water intake was not significantly affected by drinking water temperature throughout the experimental period. Rabbits receiving cooled water exhibited consumption patterns comparable to those offered water at ambient temperature, indicating that moderate cooling of drinking water did not alter voluntary water intake under the heat stress conditions evaluated.

Previous studies have reported inconsistent effects of drinking water temperature on water intake in rabbits exposed to high-ambient temperatures. Reductions in water intake have been observed when cooled water was supplied at very low temperatures (approximately 10–15 °C), particularly under conditions of intense thermal stress (THI > 30 with 35–38 °C) [11]. In contrast, other studies have reported no changes in water consumption when rabbits were provided with cooled drinking water within a moderate temperature range, similar to that applied in the present study [13,17]. These contrasting findings suggest that the effect of water temperature on water intake depends on both the degree of cooling and environmental conditions. Rabbits regulate water consumption mainly in response to hydration needs rather than using drinking behavior as a primary thermoregulatory strategy [14].

### 4.2. Physiological and Thermoregulatory Responses

The physiological responses observed in the present study indicate that cool drinking water influenced thermoregulatory processes in growing rabbits exposed to heat stress, even in the absence of productive improvements. Body temperature responses differed between morning and afternoon measurements, highlighting the importance of evaluating physiological variables in relation to the daily thermal load.

During the morning period, ear, body surface, and rectal temperatures were primarily influenced by the experimental period, reflecting temporal changes in environmental conditions. Drinking water temperature did not affect these variables, and no treatment × period interaction was detected. This response suggests that thermal conditions during the morning were not sufficiently challenging to elicit differential physiological responses between treatments, and that rabbits were able to maintain thermal homeostasis regardless of treatment. Nevertheless, a tendency for a Water × Period interaction was observed for body surface temperature (*p* = 0.10), suggesting that treatment-related differences may begin to emerge as thermal conditions intensify across periods, even though morning thermal load was insufficient to elicit physiological treatment-related effects.

In contrast, during the afternoon, when environmental temperature and THI reached their highest values, rectal temperature was significantly affected by drinking water temperature and by the treatment × period interaction. Rabbits receiving cool drinking water exhibited lower rectal temperatures during periods of greater thermal challenge, indicating a more effective short-term regulation of core body temperature under peak heat load conditions. Although the magnitude of the reduction was modest, this response is biologically relevant, as small changes in rectal temperature in rabbits have been associated with meaningful improvements in thermal balance [20].

Pearson’s correlation analysis provided a descriptive assessment of the relationship between environmental heat load and body temperature responses across experimental periods. Overall, the stronger correlations observed for ear and body temperatures compared with rectal temperature support the greater sensitivity of peripheral thermal measures to changes in THI, whereas the lower correlation values for rectal temperature are consistent with its tighter physiological regulation. Although morning and afternoon measurements showed similar general patterns, rectal temperature tended to exhibit lower and occasionally negative associations with THI during the afternoon, indicating a reduced coupling between central temperature and environmental conditions relative to peripheral temperatures. The lower correlation values observed for rectal temperature under the cool drinking water treatment in specific periods suggest a modulatory effect of water temperature on the relationship between THI and central temperature; however, this pattern should be interpreted with caution, given the narrow physiological range of rectal temperature and the limited number of observations.

The lack of treatment effects on ear and body surface temperatures suggests that the primary thermoregulatory benefit of cool drinking water occurred internally rather than through enhanced peripheral heat dissipation. In rabbits, the auricular region plays a central role in heat loss through vasodilation; however, when environmental temperatures remain high, peripheral mechanisms alone may be insufficient to reduce core temperature. Under these conditions, the ingestion of cooler water may contribute to internal heat exchange, facilitating the dissipation of excess body heat without necessarily altering surface temperature patterns. Similar reductions in rectal temperature associated with the use of cool drinking water have been reported in previous studies conducted under heat stress conditions [13,21,22]. Together, these findings indicate that drinking water temperature can modulate physiological responses to heat stress, even when growth performance remains unaffected.

The THI results provide essential context for interpreting the physiological responses observed in this study. The absence of differences in morning THI among experimental periods indicates relatively stable thermal conditions during this time of day, which is consistent with the lack of treatment effects on physiological variables measured in the morning. In contrast, the significant variation observed in afternoon and maximum THI across periods, particularly the peak values recorded during the third period, reflects a progressive intensification of thermal challenge over the course of the experiment. This pattern supports the notion that the physiological effects of cool drinking water become evident only when environmental heat load exceeds a critical threshold, explaining the significant treatment effects on rectal temperature.

The reduction in rectal temperature observed during periods of peak thermal load indicates a localized thermoregulatory benefit associated with the provision of cool drinking water under heat stress conditions. However, this physiological response was not accompanied by improvements in growth performance, and feed efficiency was reduced over the overall experimental period. Although digestive physiology was not directly evaluated in the present study, the higher feed-to-gain ratio observed in rabbits receiving cooled drinking water suggests that thermoregulatory benefits may have been accompanied by subtle metabolic or digestive costs. The ingestion of cold water may increase the energetic demand required to restore ingesta temperature to core body temperature, potentially diverting energy from productive processes [19]. Therefore, these responses should be interpreted as indicative of short-term thermoregulatory support rather than evidence of broader improvements in productive efficiency.

Although the present study demonstrates that moderate cooling of drinking water can support short-term thermoregulatory responses under heat stress, future research evaluating longer experimental periods and the integration of additional cooling strategies may help to further characterize the potential benefits of heat stress mitigation in rabbit production systems.

## 5. Conclusions

The results of this study indicate that the provision of cool drinking water under conditions of high environmental heat load induces measurable physiological responses in growing rabbits, particularly a reduction in core body temperature during the afternoon.

These effects were observed in the absence of detectable changes in live weight gain, dry matter intake, or carcass traits. However, feed-to-gain ratio over the overall experimental period was higher in rabbits receiving cooled drinking water, indicating reduced feed efficiency compared with rabbits receiving water at normal-temperature.

Under the environmental conditions evaluated, moderate cooling of drinking water acted primarily as a short-term thermoregulatory support rather than as a strategy to enhance productive performance. Overall, the findings suggest that the use of cool drinking water represents a practical and easily implementable management option to support thermoregulatory responses in rabbit production systems exposed to elevated ambient temperatures, particularly in dry tropical climates.

## Figures and Tables

**Table 1:** Morning, afternoon, and maximum THI values recorded during the experimental period.

	Period					*p*-Value		
THI ^1^	Week 1	Week 2	Week 3	Week 4	SEM	Period	Linear	Quad
Morning THI	28.27	29.04	29.50	28.53	0.42	0.19	0.51	0.04
Afternoon THI	31.44 ^c^	32.81 ^b^	33.59 ^a^	32.29 ^bc^	0.30	<0.01	0.02	<0.01
THI Max	31.99 ^c^	32.95 ^bc^	33.83 ^a^	32.54 ^bc^	0.29	<0.01	0.06	<0.01

^1^, THI = temperature–humidity index. Different lowercase letters within the same row indicate significant differences among periods (*p* < 0.05).

**Table 2:** Descriptive summary of drinking water temperature treatments.

Water ^1^	*n* ^2^	Mean (°C)	SD ^3^	CV (%) ^4^
Normal	28	33.9	1.5	4.4
Cool	28	16.7	1.8	10.8

^1^, Treatments, Normal = drinking water at ambient temperature; Cool = cool drinking water at 16.7 ± 1.8 °C. ^2^ n = One observation per treatment per day; ^3^ SD = standard deviation; ^4^ CV = coefficient of variation.

**Table 3:** Effect of drinking water temperature on the growth performance of rabbits.

	Drinking Water Temperature ^1^			
Variable ^2^	Normal	Cool	SEM ^3^	*p*-Value
Initial LW, g	1415	1443	40.1	0.63
LW, d-14, g	1804	1766	40.1	0.53
LW, d-28, g	2032	1973	40.1	0.33
DMI, d 1–14	96.90	90.38	5.60	0.46
DMI, d 15–28	92.75	88.06	2.30	0.25
DMI, d 1–28	94.80	89.23	3.60	0.35
ADG, d 1–14, g	27.73	23.04	2.48	0.27
ADG, d 15–28, g	16.32	14.80	1.05	0.38
ADG, d 1–28, g	22.03	18.92	0.93	0.10
F:G ratio, d 1–14	3.13	3.73	0.30	0.13
F:G ratio, d 15–28	5.55	5.55	0.30	0.99
F:G ratio, d 1–28	3.98	4.40	0.30	0.03

^1^, Treatments, Normal = drinking water at ambient temperature; Cool = cool drinking water at 16.7 ± 1.8 °C. ^2^, LW = live weight; DMI = dry matter intake; ADG = average daily gain; F:G = feed-to-gain; ^3^, SEM = standard error of means.

**Table 4:** Effect of drinking water temperature on the carcass traits of rabbits.

	Drinking Water Temperature ^1^			
Variable ^2^	Normal	Cool	SEM ^3^	*p*-Value
HCW, g	1079.00	1060.00	32.30	0.705
CCW, g	1068.38	1046.63	32.54	0.669
Dressing, %	52.97	53.70	0.69	0.507

^1^, Treatments, Normal = drinking water at ambient temperature; Cool = cool drinking water at 16.7 ± 1.8 °C. ^2^, HCW = hot carcass weight; CCW = cold carcass weight; ^3^, SEM = standard error of means.

**Table 5:** Effect of drinking water temperature on the water intake of rabbits.

	Drinking Water Temperature ^1^			
Water Intake	Normal	Cool	SEM ^2^	*p*-Value
Week 1	250.56	239.91	21.83	0.625
Week 2	217.55	195.36	21.83	0.313
Week 3	215.08	207.37	21.83	0.723
Week 4	215.65	196.72	21.83	0.388

^1^, Treatments, Normal = drinking water at ambient temperature; Cool = cool drinking water at 16.7 ± 1.8 °C. ^2^, SEM = standard error of means.

**Table 6:** Effect of drinking water temperature on the morning corporal temperatures of rabbits.

		Period					*p*-Value		
	Water ^1^	Week 1	Week 2	Week 3	Week 4	SEM ^2^	Water	Period	Water × Period
Ear	Normal	38.69	39.33	39.68	39.40	0.19	0.36	<0.01	0.99
	Cool	38.91	39.51	39.84	39.62				
Body	Normal	36.29	36.14	36.66	36.35 *	0.07	0.27	<0.01	0.10
	Cool	36.27	36.06	36.72	36.10 *				
Rectal	Normal	39.24	39.06	39.36	39.29	0.11	0.93	0.24	0.16
	Cool	39.22	39.29	39.31	39.17				

^1^, Treatments, Normal = drinking water at ambient temperature; Cool = cool drinking water at 16.7 ± 1.8 °C. ^2^, SEM = standard error of means. Statistical significance was declared at *p* ≤ 0.05 (**), whereas values of 0.05 < *p* ≤ 0.10 (*) were considered indicative of statistical trends.

**Table 7:** Effect of drinking water temperature on the afternoon corporal temperatures of rabbits.

		Period					*p*-Value		
	Water ^1^	Week 1	Week 2	Week 3	Week 4	SEM ^2^	Water	Period	Water × Period
Ear	Normal	41.21	41.83	42.00	41.23	0.13	0.93	<0.01	0.36
	Cool	41.04	41.8	41.98	41.35				
Body	Normal	38.48	39.34	40.29	38.76	0.09	0.58	<0.01	0.86
	Cool	38.45	39.34	40.16	38.71				
Rectal	Normal	40.50	40.25	40.20	40.22 **	0.09	0.04	<0.01	0.03
	Cool	40.41	40.00	40.00	39.76 **				

^1^, Treatments, Normal = drinking water at ambient temperature; Cool = cool drinking water at 16.7 ± 1.8 °C. ^2^, SEM = standard error of means. Statistical significance was declared at *p* ≤ 0.05 (**), whereas values of 0.05 < *p* ≤ 0.10 (*) were considered indicative of statistical trends.

**Table 8:** Pearson correlations between THI and body temperatures during the morning.

Water ^1^	Temperature	Week 1	Week 2	Week 3	Week 4
Normal	Ear	0.72	0.29	0.05	0.41
	Body	0.72	0.53	0.39	0.54
	Rectal	0.36	0.62	0.49	0.74
Cool	Ear	0.80	0.20	0.20	0.44
	Body	0.60	0.48	0.27	0.62
	Rectal	0.58	−0.24	0.51	0.28

^1^, Treatments, Normal = drinking water at ambient temperature 33.9 ± 1.5; Cool = cool drinking water at 16.7 ± 1.8 °C.

**Table 9:** Pearson correlations between THI and body temperatures during the afternoon.

Water ^1^	Temperature	Week 1	Week 2	Week 3	Week 4
Normal	Ear	0.76	0.15	0.38	0.66
	Body	0.93	0.15	0.65	0.85
	Rectal	0.54	0.12	−0.28	0.12
Cool	Ear	0.75	0.32	0.47	0.48
	Body	0.91	0.37	0.68	0.85
	Rectal	0.58	−0.09	−0.02	0.15

^1^, Treatments, Normal = drinking water at ambient temperature 33.9 ± 1.5; Cool = cool drinking water at 16.7 ± 1.8 °C.

## Data Availability

The raw data supporting the conclusions of this article will be made available by the authors on request.

## Abbreviations

The following abbreviations are used in this manuscript:

THI	Temperature–humidity index
db°C	Dry-bulb temperature
RH	Relative humidity
CV	Coefficient of variation
SD	Standard deviation
SEM	Standard error means
F:G	Feed-to-gain ratio
HCW	Hot carcass weight
CCW	Cold carcass weight
